# Efficacy of Whole-Body Vibration on Scapular Muscle Activation Pattern and Latency Timing in Modified Push-Up Position in Overhead Athletes: A Randomized Control Trial

**DOI:** 10.3390/healthcare14091237

**Published:** 2026-05-04

**Authors:** Sana Saifi, Ishant Kumar Arora, Nitin Kumar Arora, Khushi Sharma, Saurabh Sharma

**Affiliations:** 1 Center for Physiotherapy and Rehabilitation Sciences, Jamia Millia Islamia University, New Delhi 110025, India; sanasaifi14@gmail.com (S.S.); ishantarorajmi@gmail.com (I.K.A.); 2 Division of Physiotherapy, Department für Pflege-, Hebammen und Therapiewissenschaften, Bochum University of Applied Sciences, 44801 Bochum, Germany; nitin.arora@hs-bochum.de; 3 Amar Jyoti Institute of Physiotherapy, University of Delhi, Delhi 110092, India; khushisa0802@gmail.com

**Keywords:** whole-body vibration, scapular dyskinesis, shoulder injury prevention, overhead athletes, shoulder pain

## Abstract

**Highlights:**

- WBV-assisted push-up training significantly increased the activation of the serratus anterior, and lower trapezius, with the greatest improvement observed in the serratus anterior.
- Despite enhanced muscle activation, WBV training did not produce significant changes in scapular muscle onset latency compared with conventional push-up training.

**Abstract:**

**BACKGROUND:** Overhead athletes are at increased risk of shoulder dysfunction due to repetitive, high-velocity movements that can disrupt scapular muscle activation patterns. Whole-body vibration (WBV) has been proposed as a training modality to enhance neuromuscular activation, but its effects on scapular muscle activity and activation timing remain unclear. **METHODS:** This randomized controlled trial investigated the effects of WBV-assisted push-up training on scapular muscle activation and onset latency in university-level overhead athletes. Forty participants were randomly assigned to a WBV group or a control group performing identical push-up exercises without vibration for four weeks. Surface electromyography was used to assess normalized muscle activation (%MVIC) and activation latency of the upper trapezius (UT), serratus anterior (SA), and lower trapezius (LT) before and after the intervention. A 2 × 2 mixed-model ANOVA was applied for statistical analysis. **RESULTS:** Significant time × group interactions were found for muscle activation in LT and SA (*p* < 0.01). The WBV group demonstrated substantially greater increases in activations in these muscles compared with the control group, with the largest improvements observed in the serratus anterior. No statistically significant between-group differences were identified for muscle onset latency (*p* > 0.05). **CONCLUSIONS:** Adding WBV to push-up training significantly enhances key scapular muscle activation in overhead athletes but does not significantly affect muscle onset latency. WBV-assisted push-ups may act as a practical, low-load strategy to improve scapular muscle recruitment and potentially reduce the risk of sports-related shoulder injuries and pain in overhead athletes.

## 1. Introduction

Overhead athletes frequently develop alterations in scapular kinematics and neuromuscular activation patterns due to repetitive high-velocity shoulder motions, predisposing them to fatigue, instability, and overuse injuries [1,2]. Shoulder pain is highly prevalent among overhead athletes, with up to one-third experiencing symptoms during their career and many developing chronic scapular dyskinesis or rotator cuff tendinopathy [3,4]. The primary risk factors for shoulder injuries in throwers are repetitive overuse and deconditioning [5]. Furthermore, the scapulohumeral rhythm is also altered due to soft tissue derangement and injury around the shoulder [6,7], leading to scapular dyskinesia during the dynamic motions [8].

The correct muscle recruitment timing is a critical component for the optimal functioning of the scapulohumeral rhythm, along with coordination between the prime movers and the stabilizing muscles around the shoulders [9]. Scapular muscles’ preactivation helps in protecting against glenohumeral joint injuries by preserving the normal scapulohumeral rhythm [10]. Scapular muscle dysfunction has been consistently identified as an important contributing factor to shoulder pain and injury in overhead athletes.

Strength-training programs are a crucial component for athletes to prevent and rehabilitate injuries. Push-ups are commonly used in sports rehabilitation and conditioning programs to improve upper-extremity strength, scapular stability, and neuromuscular control, which are essential components in the prevention and management of shoulder injuries and pain in overhead athletes [11,12]. The serratus anterior, upper trapezius, and lower trapezius showed an increase in activation during modified push-up exercise training [12,13].

Likewise, whole-body vibration (WBV) has gained popularity in elite sports due to its effects on muscular strength in athletic individuals [14,15]. A strength-training program combined with WBV produces significant improvement in muscular strength [16]. WBV also improves flexibility [17], balance [18], and muscular blood flow [19]. The performance improvement by WBV is due to the tonic vibration reflex (TVR) that increases the sensitivity of the muscle spindle afferents. This ultimately leads to a rapid stretch-shortening cycle, shear stress on bones, and hormonal changes [14,20,21].

Surface electromyography (sEMG) is commonly used to evaluate neuromuscular responses during both exercise and whole-body vibration (WBV). Research has shown that static and dynamic exercises performed on a WBV platform can produce significant immediate and long-term increases in muscle activation [22]. Only limited research has examined at the effects of WBV on upper-body muscles, but some findings show that using vibrating dumbbells can raise neural activity in the biceps brachii [14,23]. Altered activation patterns of the serratus anterior and trapezius muscles may lead to scapular dyskinesis, impaired force transfer, and increased mechanical stress on the rotator cuff and glenohumeral joint [8]. These neuromuscular deficits can compromise dynamic shoulder stability during high-velocity overhead movements such as throwing, serving, or smashing, thereby increasing the risk of overuse injuries. Because there is limited research on the effect of WBV on EMG activity in upper-extremity muscles [23,24] and the neuromuscular latency of scapular muscles [25], there was a need to investigate its effects on muscle activation patterns and timing in this region.

The purpose of this study was to examine the effects of WBV applied during push-up exercises on scapular muscle activation amplitude and neuromuscular latency in overhead athletes. These factors are closely associated with shoulder stability and the risk of sports-related injuries. It was hypothesized that WBV-assisted push-ups would elicit greater scapular muscle activation and reduced activation latency compared with conventional push-up exercises.

## 2. Methods

**Trial Design**

A parallel-group randomized controlled trial was conducted to examine the effects of combined whole-body vibration (WBV) and push-up training on scapular muscle activation and temporal recruitment patterns in overhead athletes. Participants were assigned in a 1:1 ratio to either a WBV intervention group or a control group performing identical exercises without vibration. All assessments were performed at baseline and after the four-week intervention period. CONSORT 2025 checklist for randomised control trials is available in Supplementary Data A.

**Ethical consideration**

The study was approved by the Institutional Ethics Committee of Jamia Millia Islamia, New Delhi, India (IEC reference: 26/11/270/JMI/IEC/2019), approved on 26 November 2019, and was registered with the Clinical Trials Registry–India (CTRI/2020/11/029240); registered on 23 November 2020. Written informed consent was obtained from all participants prior to enrollment.

**Trial setting**

All participants were recruited using a convenience sampling method from university-level overhead athletes at the Nawab Mansur Ali Khan Pataudi Sports Complex, Jamia Millia Islamia, New Delhi, India. Each participant was actively involved in competitive overhead sports such as volleyball, badminton, tennis, or bowling, and the dominant shoulder was assessed for this study. All assessments and intervention procedures were carried out in a research laboratory at the Center for Physiotherapy and Rehabilitation Sciences, Jamia Millia Islamia University.

**Eligibility criteria**

Inclusion criteria: University-level overhead athletes with full shoulder range of motion, no history of shoulder pain in the past six months, and the ability to understand instructions in English.

Exclusion criteria: Participants with any current or recent upper-extremity injury, history of rotator cuff injury within the past year, previous shoulder surgery, metabolic or neuromuscular disorders, spinal postural abnormalities within the last six months, prior exposure to WBV training within the last three months, or history of epilepsy or seizures were excluded.

**Intervention and comparator**

All participants completed a familiarization session with the training procedures before starting the study. After random allocation into two groups (Group A and Group B), participants received their respective training interventions.

Group A performed push-ups on a whole-body vibration (WBV) platform. Following the protocol described by Ashnagar et al. [24], athletes adopted a modified push-up position with hands placed shoulder-width apart and equidistant on the WBV platform, elbows slightly flexed, and knees on the floor. Participants were instructed to perform elbow flexion through either a full or self-selected range of motion, followed by immediate extension [26]. The training program consisted of 4 weeks of WBV-assisted push-ups, performed three times per week, with each session including three sets of ten repetitions. Each session began with a 5 min warm-up comprising light dynamic movements. WBV parameters were set in accordance with previous research, with an amplitude of 5 mm and a frequency of 30 Hz [14,26,27]. Push-ups were performed at a controlled pace (approximately 2 s eccentric, 1 s pause, and 2 s concentric), with a 1 min rest interval between sets. The protocol was maintained at a fixed dosage throughout the 4-week period to isolate the neuromuscular effects of WBV from progressive strength-training adaptations. Figures of WBV intervention are available in Supplementary Data B.

Group B (comparator group) performed push-ups on a flat, non-vibrating surface following the same training duration (4 weeks), weekly frequency (three sessions per week), session volume (three sets of ten repetitions), warm-up procedures, and controlled tempo as Group A, but without vibration exposure. The detailed intervention protocol is presented in Table 1 and is described in accordance with the Consensus on Exercise Reporting Template (CERT) [28] to enhance the replicability of the findings.

**Outcomes**

Pre- and post-exercise testing were carried out using the same standardized procedures and in the same environment. The initial data collection took place on each athlete’s first day of participation, during which a detailed history was obtained, including the type of overhead sport, years of experience, dominant arm, competitive level, and relevant medical background. Surface electromyography was used as the standardized assessment tool, and all measurements were performed by the same therapist before the intervention and again after four weeks of training. All sessions were supervised by a trained therapist to ensure correct technique and adherence.

Surface electromyography

The sEMG was performed using PowerLab 15T (AD Instruments Ltd., Sydney, Australia). The electrodes were connected to a two-channel electromyography (EMG) receiver, and the placement of the electrodes was followed by the inspection of the electromyography signals on a computer screen during each specific muscle testing. The sampling rate was set at 1000, and the preamplification of all the raw myoelectric signals was performed with an overall gain of 1000, a common rate rejection ratio of 115 dB, and a signal-to-noise ratio of 1 mV RMS baseline noise. Raw EMG signals were band-pass filtered between 20 and 450 Hz using a fourth-order Butterworth filter to remove low-frequency motion artifacts and high-frequency noise. A notch filter at 50 Hz was additionally applied to eliminate power line interference. The filtered signals were subsequently full-wave rectified and smoothed using a root mean square (RMS) algorithm with a 100 ms moving window. Electrode crosstalk was minimized by strict adherence to standardized electrode placement guidelines and by verifying signal specificity during pilot testing. To warm up the scapular muscles before testing, shoulder movements were performed in all directions, the hair was shaved, and the skin was cleaned with alcohol to reduce impedance (<10 kΩ). Bipolar surface electrodes were positioned at a distance of 2 cm each over the upper, lower trapezius, and serratus anterior and the posterior deltoid, with a reference electrode placed at the ipsilateral clavicle (Table 2). The muscle activity of the upper trapezius (UT), serratus anterior (SA), and lower trapezius (LT) of the dominant shoulder was recorded. These three scapulothoracic muscles were examined because they are considered to be the key muscles that form force couples, helping to control scapular position and motion [29]. The neuromuscular onset latency of these muscles was then analyzed relative to the posterior deltoid onset timing [30]. Figures of EMG electrode placement are available in Supplementary Data B.

Maximum Voluntary Isometric Contractions (MVICs)

The participants performed three maximal voluntary contractions (MVIs) of the upper trapezius, lower trapezius, and serratus anterior against the resistance given by the therapist. Table 2 provides the MVIC testing positions for each muscle. To control the duration of muscle contractions, a metronome was used, and a 5 s pause was given between contractions [1,29].

Root Mean Square (RMS)

After taking the MVICs, a 5 min rest was provided, followed by bilateral arm elevation in the scapular plane (30° anterior to the coronal plane), performed during all three phases of movement, i.e., concentric, isometric, and eccentric; each phase was maintained for 3 s, 3 s of pause was given between each trial and five trials of this movement were performed by the participants [30]. Normalization of EMG of UT, LT, and SA was performed, and then the MVIC value was represented as a percentage of the total isometric activity.

Formula of normalization (%MVIC): RMS/MVIC × 100.

Muscle Latency

The muscle contraction onset time was determined and analyzed relative to the onset timing of the posterior deltoid. For timing measurement, only the concentric phase was analyzed, and the point when the muscle activity rose above the resting activity by two standard deviations for a minimum of 50 ms was utilized [30].

**Sample Size**

A priori sample size calculation was performed using G*Power software (version 3.1.9.7; Heinrich-Heine-Universität Düsseldorf, Germany), based on a repeated-measures ANOVA (within–between interaction). The calculation assumed an alpha level of 0.05, power of 0.80, expected effect size of f = 0.40, and two groups with two measurement time points. The analysis indicated that a minimum sample size of 40 participants was required. The calculation was based on parameters reported by [24].

**Randomization**

Participants were randomly allocated to the intervention groups using a computer-generated random sequence. Allocation was concealed by an independent researcher using sealed, opaque, sequentially numbered envelopes. The randomization sequence was generated by an investigator not involved in participant recruitment or outcome assessment. Group assignments were revealed only after baseline measurements were completed. The blinding of participants and trainers was not feasible due to the nature of the intervention. However, the assessor responsible for EMG electrode placement, data acquisition, and signal processing remained blinded to group allocation throughout the study.

## 3. Statistical Methods

Data were analyzed using R Studio (v4.3.3). Descriptive statistics were expressed as the mean ± standard deviation (SD). Normality and homogeneity of variance were assessed using the Shapiro–Wilk and Levene’s tests, respectively. A 2 × 2 mixed-model ANOVA was performed to examine the main effects of time (pre vs. post), group (control vs. WBV), and their interaction on normalized muscle activation (%MVIC) and neuromuscular activation timing (latency) for each muscle. The effect sizes were reported as partial eta squared (η^2^_p_) for ANOVA effects and Cohen’s d for between-group differences. Statistical significance was set at *p* < 0.05.

## 4. Results

**Figure 1:**
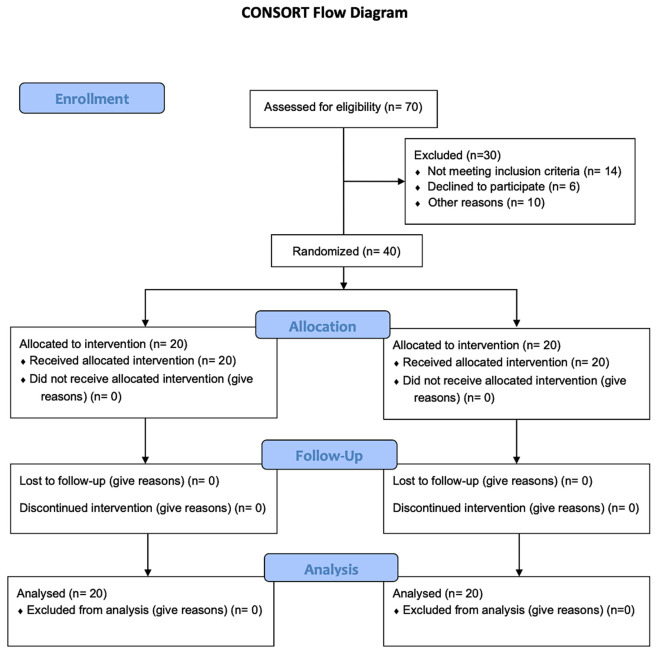
CONSORT flow chart illustrating screening, eligibility, exclusions, randomization, and final sample analyzed.

Forty overhead athletes completed the study and were included in the final analysis. Baseline characteristics were comparable between groups. No adverse events occurred during the intervention period. The progression of participants through enrollment, allocation, follow-up, and analysis is shown in the CONSORT flow diagram (Figure 1).

**Recruitment**

Forty university-level athletes participated in this study, with equal allocation to the experimental and control groups. The mean age of the experimental and control groups was 21.45 ± 1.47 and 21.40 ± 1.27 years, respectively; the mean weight was 64.6 ± 7.22 and 68.1 ± 2.61 kg; the mean height was 1.70 ± 0.06 and 1.72 ± 0.05 m; and BMI was 22.12 ± 2.02 and 23.07 ± 1.07 kg/m^2^, respectively. No significant baseline differences were found between groups as summarized in Table 3.

**Intervention and comparator delivery**

The exercise intervention was delivered by licensed physiotherapists as planned. No major deviations from the intervention protocol were reported.

**Outcomes**

Muscle activation (%MVIC)

A 2 × 2 mixed-model ANOVA revealed significant main effects of time for all the scapular muscles, including the upper trapezius (F(1,38) = 9.51, *p* = 0.0038, and η^2^_p_ = 0.200), serratus anterior (F(1,38) = 67.74, *p* < 0.001, and η^2^_p_ = 0.641), and lower trapezius (F(1,38) = 27.63, *p* < 0.001, and η^2^_p_ = 0.421), indicating overall increases in muscle activation over time.

A significant main effect of group was observed only for the serratus anterior (F(1,38) = 20.08, *p* < 0.001, and η^2^_p_ = 0.346). In contrast, no significant group effects were found for the upper trapezius (F(1,38) = 0.26, *p* = 0.616, and η^2^_p_ = 0.007) or lower trapezius (F(1,38) = 1.74, *p* = 0.195, and η^2^_p_ = 0.044).

Importantly, significant time × group interaction effects were observed for the serratus anterior (F(1,38) = 45.89, *p* < 0.001, and η^2^_p_ = 0.547) and lower trapezius (F(1,38) = 21.29, *p* < 0.001, and η^2^_p_ = 0.359), indicating that the WBV group demonstrated significantly greater improvements compared with the control group. In contrast, the interaction effect for the upper trapezius was not statistically significant (F(1,38) = 0.87, *p* = 0.357, and η^2^_p_ = 0.022), suggesting comparable changes between groups.

Between-group differences in change scores (post–pre) further supported these findings. Significant differences were observed for the serratus anterior (*p* < 0.001) and lower trapezius (*p* < 0.001), indicating substantially greater improvements in the WBV group. No significant between-group difference was observed for the upper trapezius (*p* = 0.359). The effect sizes (η^2^_p_) indicated large interaction effects for serratus anterior and lower trapezius activation, whereas the upper trapezius demonstrated only a small effect. The descriptive results are presented in Table 4.

Muscle Onset Latency

No significant main effects of time or group were observed for muscle onset latency (*p* > 0.05). Similarly, no significant time × group interaction effects were found for the upper trapezius (F(1,38) = 0.33, *p* = 0.567, and η^2^_p_ = 0.009), serratus anterior (F(1,38) = 3.44, *p* = 0.071, and η^2^_p_ = 0.083), or lower trapezius (F(1,38) = 0.07, *p* = 0.796, and η^2^_p_ = 0.002).

**Figure 2:**
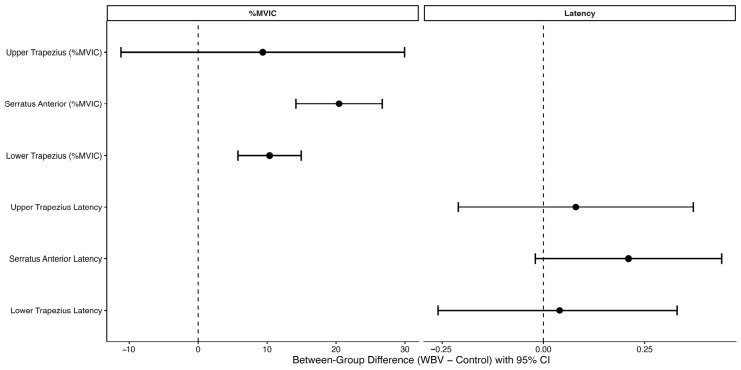
Between-group differences in change scores (post–pre) for scapular muscle activation (%MVIC) and onset latency. Positive values indicate greater improvements in the WBV group. The dashed vertical line represents no between-group difference. Confidence intervals that do not cross zero indicate statistically significant differences.

Between-group comparisons of change scores showed no statistically significant differences for upper trapezius latency (*p* = 0.570), serratus anterior latency (*p* = 0.075), or lower trapezius latency (*p* = 0.796). The between-group differences in change scores, along with their 95% confidence intervals, are illustrated in Figure 2. The mean ± SD values for latency are reported in Table 4.

Detailed statistical results, including full ANOVA tables (F-values, *p*-values, and effect sizes), confidence intervals, and between-group comparisons of change scores, are provided in the Supplementary Data C.

**Harms**

No adverse events, injuries, or discomfort related to the intervention were reported during the study period.

## 5. Discussion

Our study demonstrated that four weeks of WBV training showed marked improvements in the muscle activation of the serratus anterior and lower trapezius muscles compared with conventional push-up exercises. However, upper trapezius activation was not greater when compared with conventional push-up exercises. The serratus anterior exhibited the greatest increase in activation, suggesting the enhanced recruitment of a key scapular stabilizer involved in maintaining scapulothoracic rhythm and shoulder joint control. From a clinical perspective, these findings suggest that WBV-assisted push-up training may be an effective adjunct to rehabilitation programs to enhance scapular muscle recruitment. The improved activation of these stabilizing muscles may contribute to better shoulder joint mechanics, which may have mechanistic relevance for shoulder function. However, potential effects on injury risk or return to sport outcomes were not assessed in this study. The absence of significant changes in upper trapezius activation may be attributed to its relatively lower and less responsive activation profile during push-up tasks compared with the serratus anterior, which demonstrates greater activation and adaptability across exercise variations [13]. Moreover, WBV intervention did not produce significant improvements in the temporal recruitment of the scapular muscles. These findings are consistent with previous studies investigating the acute effects of WBV on neuromuscular latency [31]: WBV has been shown to primarily increase EMG activity, reflecting enhanced neuromuscular activation, without changes in force, strength, or muscle morphology. Despite these increases in activation, no alterations in short-latency responses were observed following WBV exposure. The improved activation of the serratus anterior and trapezius muscles is particularly relevant for sports physical therapy because these muscles play a key role in maintaining scapular stability and preventing abnormal shoulder mechanics during overhead movements.

Serratus anterior and lower trapezius activation is essential for maintaining the position of the scapula and preventing the superior migration of the humeral head during overhead movements. Deficits in scapular muscle activation have previously been associated with shoulder pain and overuse injuries in overhead athletes [9,32]. These findings provide mechanistic insights that may be relevant for the management of shoulder injuries in the athletic population, which remain a leading cause of athletes being unable to train or compete, especially in sports that involve frequent overhead movements. Shoulder conditions such as subacromial pain syndrome, rotator cuff tendinopathy, and glenohumeral instability are particularly common in volleyball, badminton, and tennis players. Although clinically distinct, these conditions share a common underlying mechanism: reduced scapular muscle stabilization during repeated, high-velocity arm movements [33,34]. The observed increases in serratus anterior and lower trapezius activation are mechanistically relevant, as these muscles contribute to scapular upward rotation and posterior tilt, both of which are associated with a reduced risk of subacromial impingement [34,35,36]. Importantly, WBV-assisted push-ups facilitate these neuromuscular gains within a low-load, closed kinetic chain framework, making them particularly suited for early-phase rehabilitation and in-season injury prevention where high-resistance loading of the shoulder complex is contraindicated [37]. From a rehabilitation perspective, incorporating WBV-assisted exercises may facilitate early neuromuscular re-education without imposing excessive mechanical load on the healing tissues, as vibration stimuli have been shown to enhance shoulder muscle activation and neuromuscular responses during exercise [25]. This is particularly relevant in clinical scenarios where pain, inflammation, or tissue healing constraints limit the use of high-resistance strengthening exercises. This suggests that WBV-assisted push-up training may be a promising and sport accessible tool in physical therapy, although its clinical effectiveness remains to be established. Prospective studies with clinical shoulder pain populations and sport-specific functional outcomes are needed to confirm whether the neuromuscular improvements demonstrated here translate into measurable reductions in injury incidence and pain burden in overhead athletes.

The present findings are consistent with the previous literature reporting an increase in neuromuscular activation following WBV-based interventions [24]. Ashnagar and Collageaus showed that WBV stimulus improved the muscular activity of the upper-limb muscles while sustaining the static modified pushup position [24]. Gyulai et al. also demonstrated that acute WBV combined with push-up exercise training influenced the dynamic work of the upper-body muscles [26]. On the contrary, WBV combined with resistance training for 10 weeks did not reveal any between-group differences, but the authors claimed that indirect strengthening of the rotator cuff was possible through WBV [38].

Grant et al. investigated neuromuscular onset latency alterations after WBV exposure on the upper-extremity muscles [25] and concluded that the use of WBV alters the timing of shoulder muscle recruitment, with the effect of improving the readiness for the movement. However, the study recorded neuromuscular onset latency immediately after vibration exposure, which could explain the preactivation of the rotator cuff as a temporary adaptation by the shoulder muscles to provide stability to the shoulder complex. Beyond the temporal limitations of measurement, the non-significant latency findings may also reflect the specificity of neuromuscular adaptation to the training context. The tonic vibration reflex, which underpins the acute neuromotor response to WBV, predominantly acts through Ia afferent pathways to increase spindle sensitivity and motor unit discharge rates during the exposure window [39]. However, converting this short-term sensory effect into lasting changes in anticipatory muscle onset, a process controlled by central feedforward motor control pathways, likely requires task-specific practice over a longer training period than four weeks [33,40]. Supporting this interpretation, Taube et al. demonstrated that corticospinal adaptations governing anticipatory postural muscle activity require a minimum of six to eight weeks of consistent neuromuscular loading to consolidate into measurable changes in premotor latency [37]. Future trials incorporating extended training durations, progressive vibration parameters, and more sensitive temporal detection methods such as high-density sEMG are warranted to more definitively characterize WBV-induced changes in scapular muscle onset timing.

Shoulder pain in overhead athletes usually presents with an altered neuromuscular pattern and a lack of scapular control [1,2]. Rehabilitation strategies targeting scapular muscle recruitment may improve clinical and performance outcomes by reducing abnormal loading on the glenohumeral joint [9]. The improvements in muscle activation observed in this study show that WBV-based exercise could be a useful adjunct in training programs aimed at preventing and rehabilitating shoulder pain in overhead sports. Clinically, WBV-assisted push-ups may offer a time-efficient and low-load strategy for clinicians working with overhead athletes, particularly in low-load training contexts. However, its effectiveness in rehabilitation or injury prevention programs requires further investigation. Future studies could evaluate the duration for which latency effects last following WBV exposure. Analyzing the muscle latency in overhead athletes and utilizing interventions that could improve latency would be beneficial for elite sports in improving the quality of sports and preventing sports-related overuse injuries.

Limitations

This study has several limitations that should be considered when interpreting the findings. First, the sample size was relatively small and limited to university-level overhead athletes, which may restrict the generalizability of the results to other populations. Second, the fixed four-week protocol without progressive load increases may have restricted the magnitude of adaptation. Finally, the blinding of participants and therapists was not feasible due to the nature of the intervention.

## 6. Conclusions

This study demonstrated that adding whole-body vibration to push-up training significantly enhanced scapular muscle activation in university-level overhead athletes compared with standard push-ups alone. These findings suggest that WBV-assisted push-ups may serve as a practical, low-load strategy to improve scapular muscle recruitment and enhance shoulder joint stability, supporting neuromuscular activation of key scapular stabilizers. While injury prevention and pain reduction were not directly assessed in this study, the enhanced scapular muscle activation observed suggests a mechanistic pathway through which WBV training may also improve scapular stability and potentially reduce the risk of shoulder pain and overuse injuries commonly observed in overhead sports. Further research is needed to examine whether different training parameters or longer interventions influence neuromuscular activation timing and injury-related outcomes.

## Figures and Tables

**Table 1:** Consensus of Exercise Template checklist for the description of the exercise protocol.

Section/Item	Checklist Requirement	Details for This Study
**WHAT: Materials**	**1. Exercise equipment**	WBV platform for experimental group; flat surface for control push-ups.
**WHO: Provider**	**2. Instructor qualifications**	All sessions supervised by the same trained therapist.
**HOW: Delivery**	**3. Individual or group**	Exercises performed individually.
	**4. Supervised or unsupervised**	Fully supervised sessions.
	**5. Adherence measurement**	Session attendance recorded; 3 sessions/week for 4 weeks.
	**6. Motivation strategies**	None provided (N/A).
	**7a. Decision rules for progression**	No progression criteria; fixed protocol.
	**7b. How progression occurred**	No progression; same sets/repetitions for 4 weeks.
	**8. Exercise description**	**Group A (WBV):** Modified push-up on WBV platform; hands shoulder-width apart, elbows slightly flexed, knees on floor; flexion/extension through full or self-selected ROM; 3 sets × 10 reps; 1 min rest; 3 sessions/week for 4 weeks. **Group B (Control):** Same push-up protocol on a flat surface without vibration.
	**9. Home program**	None.
	**10. Non-exercise components**	None.
	**11. Adverse events**	No adverse events reported.
**WHERE: Location**	**12. Training setting**	Conducted indoors at Nawab Mansur Ali Khan Pataudi Sports Complex, JMI, New Delhi.
**WHEN and HOW MUCH: Dosage**	**13. Intervention dosage**	4-week program; 3 sessions/week; 3 sets of 10 repetitions per session; 1 min rest between sets. 5 min dynamic warm-up.
**TAILORING**	**14a. Generic or tailored**	Generic exercises; same for all participants.
	**14b. Tailoring method**	No tailoring.
	**15. Starting level decision rule**	All participants began directly with the standardized protocol due to being trained athletes.
**HOW WELL**	**16a. Fidelity measurement**	Attendance monitored; supervised training ensured compliance.
	**16b. Delivery as planned**	Intervention delivered as planned with no deviations.

The bold formatting in the table is intentional for emphasis and consistency.

**Table 2:** Electrode placement and MVIC test positions.

Muscles	Electrode Placement	Action
Upper trapezius	Subject’s position: Shoulder abduction to 90°. Electrode placement: 2 cm laterally to the midpoint of the distance between the C7 spinous process and acromial tip.	Resisted shoulder abduction at 90° with head and neck contralaterally rotated and ipsilaterally side-bent.
Lower trapezius	Subject position: Shoulder flexed to 90°. Electrode placement: Obliquely placed at a point 5 cm inferior and medial to the root of scapular spine.	The subject is prone and resisting the arm raised overhead in line with the lower trapezius muscle fibers.
Serratus anterior	Subject position: Shoulder abduction to 90°. Electrode placement: Vertically at the 6th and 8th rib level along the mid-axillary line.	Shoulder flexed to 125° in the plane of the scapula against the resistance.
Posterior deltoid	Subject position: Shoulder in abduction, slight extension, and slight internal rotation. Electrode placement: 2 cm below the lateral border of the scapula with an oblique orientation, parallel to the muscle fiber direction.	This muscle was only used to calculate the muscle latency.

**Table 3:** Comparison of demographic characteristics between groups.

Participants’ Characteristics	Experimental (*n* = 20)	Control (*n* = 20)
Age (years)	21.40 (1.27)	21.45 (1.47)
Height (cm)	171.2 (4.29)	170.98 (5.54)
Weight (kg)	66.50 (4.41)	64.20 (5.41)
BMI (kg/m^2^)	22.61 (1.43)	22.03 (1.68)

Values are presented as the mean (standard deviation). cm: centimeters; kg: kilograms; BMI: body mass index; cm: centimeters; and SD: standard deviation.

**Table 4:** Comparison of the mean ± SD for the WBV and control groups before and after the treatment.

Muscle	Variable	Control Pre	Control Post	WBV Pre	WBV Post
UT	%MVIC	25.73 ± 13.93	36.53 ± 48.94	24.72 ± 13.21	44.90 ± 19.06
UT	Latency	0.29 ± 0.23	0.22 ± 0.21	0.27 ± 0.65	0.28 ± 0.51
SA	%MVIC	19.31 ± 7.28	21.51 ± 7.79	24.95 ± 13.81	47.58 ± 16.96
SA	Latency	0.27 ± 0.20	0.21 ± 0.35	0.14 ± 0.63	0.29 ± 0.33
LT	%MVIC	13.42 ± 7.41	14.14 ± 8.94	12.62 ± 10.06	23.70 ± 15.95
LT	Latency	0.35 ± 0.30	0.32 ± 0.45	0.07 ± 0.78	0.07 ± 0.43

Abbreviations: %MVIC UT: percentage maximum voluntary isometric contraction of upper trapezius; %MVIC LT: percentage maximum voluntary isometric contraction of lower trapezius; %MVIC SA: percentage maximum voluntary isometric contraction of serratus anterior; UT Latency: upper trapezius latency; LT Latency: lower trapezius latency; SA Latency: serratus anterior latency; and significant difference = *p* ≤ 0.05. Mean ± SD: Mean and standard deviation.

## Data Availability

The datasets generated and/or analyzed during the current study are not publicly available because of the ethical restrictions related to human participant data. The de-identified minimal datasets that support the findings of this study (i.e., anonymized participant characteristics and electromyographic outcome variables used in the statistical analyses) are available from the corresponding author upon reasonable request.

## Supplementary Materials

The following supporting information can be downloaded at: https://www.mdpi.com/article/10.3390/healthcare14091237/s1 Supplementary Data A: CONSORT 2025 checklist for randomised control trials. Supplementary Data B: Figures for modified push-up position on the whole-body vibration (WBV) platform and EMG electrode placement for upper trapezius, lower trapezius, serratus anterior, and posterior deltoid muscles (Figures S1–S3). Supplementary Data C: Data analysis tables, including: Table S1: Between-group differences showing mean difference, confidence intervals, *p*-values, and Cohen’s d for WBV and control groups Table S2: Mixed ANOVA results showing F values, *p*-values, and effect sizes for time, group, and interaction effects.
